# Dysosmia and dysgeusia associated with duloxetine

**DOI:** 10.1136/bcr-2017-222470

**Published:** 2017-11-23

**Authors:** Katsuyuki Yoshida, Takahiko Fukuchi, Hitoshi Sugawara

**Affiliations:** 1 Division of General Medicine, Department of Comprehensive Medicine 1, Saitama Medical Center, Jichi Medical University, Saitama, Saitama, Japan; 2 Division of General Medicine, Department of Comprehensive Medicine 1, Saitama Medical Center, Jichi Medical University, Saitama, Saitama, Japan; 3 Division of General Medicine, Department of Comprehensive Medicine 1, Saitama Medical Center, Jichi Medical University, Saitama, Saitama, Japan

**Keywords:** Pharmacology And Therapeutics, Unwanted Effects / Adverse Reactions

## Abstract

Common adverse effects of serotonin–norepinephrine reuptake inhibitors are nausea, dry mouth, dizziness and headache. We describe the case of a patient with dysosmia and subsequent dysgeusia associated with duloxetine. A 68-year-old Japanese woman with a history of type 1 diabetes mellitus, hypertension, insomnia and reflux esophagitis presented to a local hospital with bilateral leg pain; she was treated with duloxetine. However, after 4 weeks, she sensed rotten egg smell, experienced nausea and vomiting and was admitted to our hospital. We diagnosed dysosmia using the T&T olfactometer threshold test and dysgeusia using filter paper disk method. Taste was assessed using electrogustometry. We suspected that dysosmia and dysgeusia were adverse effects of duloxetine. After stopping duloxetine, her symptoms gradually subsided and the above test results improved, despite continuing the other ongoing medication. To the best of our knowledge, this is the first case report of dysosmia and dysgeusia associated with duloxetine.

## Background

Serotonin–norepinephrine reuptake inhibitors (SNRI) are used to treat depressive disorders and certain types of chronic pain.^1^ The most common adverse effects of SNRIs are nausea, dry mouth, dizziness and headache.^2^ It remains unknown which drug is likely to cause drug-related concomitant taste and smell dysfunction.^3–5^ Here we describe distortion of taste (dysosmia) and smell (dysgeusia) as new adverse effects of duloxetine.

## Case presentation

A 68-year-old Japanese woman with medical histories of type 1 diabetes mellitus, hypertension, insomnia and reflux esophagitis presented to a local hospital with bilateral leg pain due to diabetic neuropathy and was treated with duloxetine. After 4 weeks, she vomited blood and was admitted to our hospital for further investigation. Prior to hospitalisation, she reported a 4-day history of a rotten egg smell, vomiting and an inability to eat. She described that she experienced the smell for the first time when she visited a coffee shop. She had no history of smoking, head trauma, allergic rhinitis or upper respiratory tract infection before the onset of symptoms and also showed no symptoms of chronic or acute recurrent rhinosinusitis or rhinitis.

Her medication included long-acting insulin analogue (glargine) 14 units at bed time, rapid-acting insulin analogue (aspart) 8 units each before meals, amlodipine (5 mg/day), lansoprazole (15 mg/day), brotizolam (0.25 mg/day) and duloxetine (20 mg/day).

Physical examination revealed a body mass index of 20.5 kg/m^2^, temperature of 37.3°C, pulse of 127 beats/min, blood pressure of 184/100 mm Hg, respiratory rate of 20 breaths/min and oxygen saturation of 96% in room air. She was alert and oriented, with no evidence of dementia. Her mouth was dry, but her capillary refill time was <2 s. There was no sinus or abdominal tenderness. Neurological findings were normal, with no evidence of tremor at rest, rigidity and postural instability.

## Investigations

The laboratory data were as follows: white cell count, 10.28x10^9^/L; haemoglobin, 15 g/dL; platelets, 25.9×10^4^/μL; haemoglobin A1c, 5.9%; casual plasma glucose level, 244 mg/dL; blood urea nitrogen, 34 mg/dL; creatinine, 0.49 mg/dL; potassium, 2.9 mmol/L; zinc, 49 µg/dL (65–110); and copper, 128 µg/dL (76–141). Upper gastrointestinal endoscopy showed an oesophageal erosive lesion but no bleeding or obstruction. Head MRI showed fluid intensity in the maxillary sinus but no atrophy of the hippocampus or diffuse changes in the temporal and frontal lobes.

**Figure 1:**
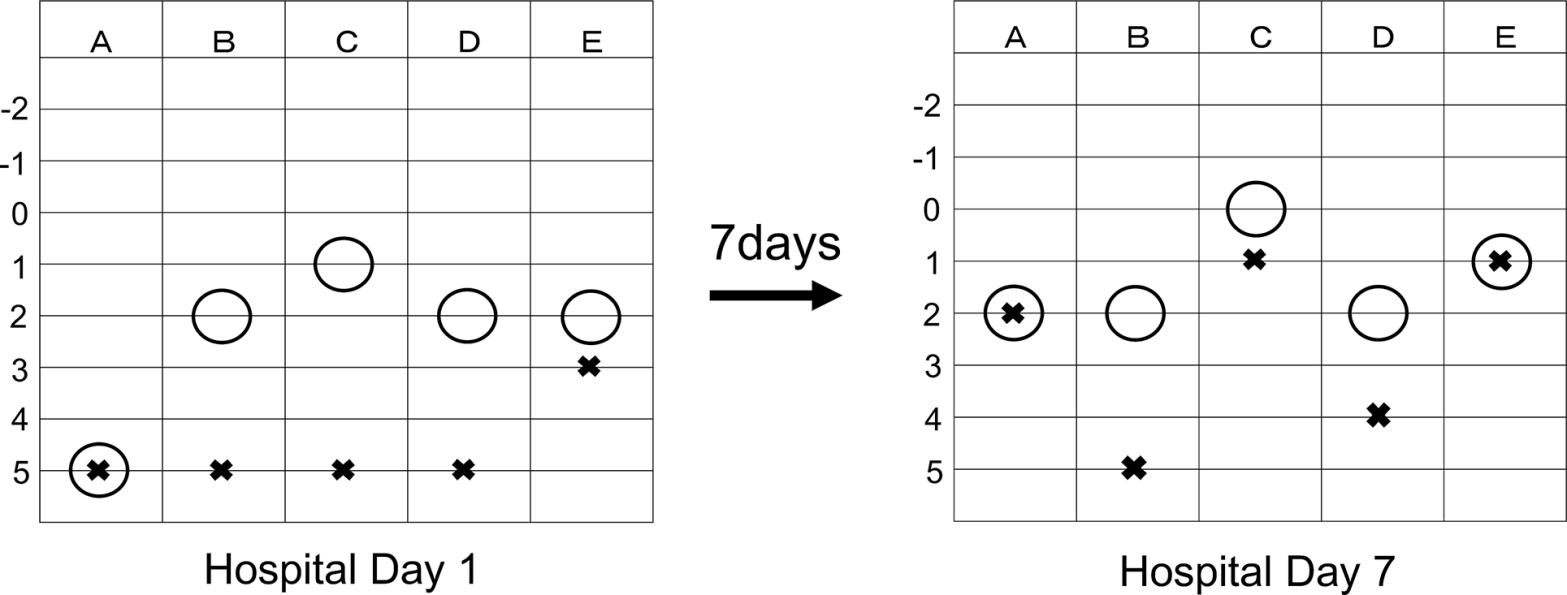
T&T olfactometer threshold test on admission day (left) and 7 days after discontinuing duloxetine (right). Alphabets represent specific odours: A, rose; B, caramel; C, rotten egg; D, sweet; and E, faeces. Range: −2 to 5, lower number indicates better smell threshold. Circle indicates the smell detection threshold. Saltire indicates the smell recognition.

Olfactory acuity tests were performed using the T&T olfactometer threshold test (figure 1), which showed that olfactory detection thresholds were <2, except those for the rotten egg smell, and all olfactory recognition thresholds were <3. The filter paper disk method was used for taste assessment, which indicated that recognition levels for sweet, salt, sour and bitter tastes were <4 (table 1). Electrogustometry (table 2) of the areas of the chorda tympani and glossopharyngeal and major petrosus nerves showed that taste recognition levels were all bilaterally >10 dB.

**Table 1:** Filter paper disk method for taste assessment on admission day (HD 1) and 7 days after discontinuing duloxetine (HD 7)

Nerve		Taste							
		Sweet		Salt		Sour		Bitter	
		HD 1	HD 7	HD 1	HD 7	HD 1	HD 7	HD 1	HD 7
Chorda tympani	Right	VI	II	V	III	VI	I	V	III
	Left	V	IV	VI	III	V	II	VI	IV
Glossopharyngeal	Right	VI	III	VI	III	V	V	V	II
	Left	V	V	V	II	V	III	VI	VI
Major petrosus	Right	VI	VI	VI	VI	VI	VI	VI	VI
	Left	VI	VI	VI	VI	VI	VI	VI	V

Roman numbers indicate taste concentration (ranges I–V); lower number indicates lighter. VI means no recognition of the taste.

HD, hospital day.

**Table 2:** Electrogustometry on admission day (HD 1) and 7 days after discontinuing duloxetine (HD 7)

Nerve		Decibel	
		HD 1	HD 7
Chorda tympani	Right	10	−6
	Left	20	−4
Glossopharyngeal	Right	20	8
	Left	20	6
Major petrosus	Right	>34	34
	Left	>34	30

Values indicate taste detection threshold. Lower value indicates better detection threshold.

HD, hospital day.

## Differential diagnosis

The patient showed both smell and taste disorders. Using the history of the patient and laboratory, physiological and imaging findings, the differential diagnosis for the taste disorder included adverse drug effects of duloxetine started before the onset of dysgeusia or the other ongoing medication, zinc deficiency, sinusitis and diabetic neuropathy, and that for the smell disorder included adverse drug effects of duloxetine started before the onset of dysosmia or the other ongoing medication, sinusitis and diabetic neuropathy.

## Treatment

Soon after stopping duloxetine, both smell and taste disorders improved (figure 1 and tables 1 and 2), despite continuing the other ongoing medication.

## Outcome and follow-up

Seven days after discontinuing duloxetine (hospital day 7), olfactory acuity tests showed improved recognition levels of rose, rotten egg and faeces smells of >3. Taste assessment using the filter paper disk method showed that the recognition level was partially improved by >3 and that using electrogustometry showed that the detection level was improved by >10 dB in the areas of the chorda tympani and glossopharyngeal nerve. If the other differential diagnosis was correct, then the symptom would not have improved only by stopping duloxetine.

After discharging from our hospital, her olfactory and taste disorders have not recurred so far.

## Discussion

This report indicates that duloxetine caused dysosmia and subsequent dysgeusia because both smell and taste disorders reversibly recovered 7 days after discontinuing duloxetine, despite continuing the other ongoing medication. This clinical course between the discontinuation of duloxetine and improvement of taste and smell sensation provides objective evidence to support duloxetine as the cause of these adverse effects. Two important clinical issues arise from the clinical course of the present study patient. The first is whether these disorders have already been reported as adverse effects of duloxetine, and the second concerns the mechanism by which duloxetine or by which drug interactions with duloxetine cause these disorders.

The primary causes of olfactory dysfunction are allergic and viral rhinitis, influenza, head trauma, dementia of the Alzheimer type, Parkinson’s disease, diabetes mellitus and malnutrition.^3^ Although this patient had several years’ history of type 1 diabetes mellitus, hypertension, insomnia, reflux esophagitis, and chronic bilateral maxillary sinusitis and low serum zinc levels, which were found at this admission, there had been no problem with her sense of smell before taking duloxetine, and her sense of smell recovered soon after discontinuing duloxetine, despite continuing the other ongoing medication. This clinical course proved that duloxetine was the major cause of dysosmia and subsequent dysgeusia in this patient.

Although the incidence of drug-induced taste and smell disorders is quite high, there are few reports available in the literature regarding their true incidence.^3–5^ Thus, the true incidence of drug-induced taste and smell disorder may possibly be underestimated. Available evidence from two reviews^3 4^ and a report of an Italian database^5^ indicates that calcium channel blockers (amlodipine, nifedipine, diltiazem), doxazosin, ACE inhibitors (enalapril, ramipril), enalapril maleate-felodipine, enalapril maleate-hydrochlorothiazide, tocainide, amiodarone, statins (lovastatin, atorvastatin), fluoroquinolones (moxifloxacin, levofloxacin), macrolides (clarithromycin, azithromycin, roxithromycin), amoxicillin/clavulanate, terbinafine, beclomethasone and methotrexate impair the sense of both smell and taste. We specifically searched the PubMed database for literature published in English and Japanese until July 2017 to identify eligible articles that simultaneously met the Medical Subject Headings terms ‘duloxetine hydrochloride’ and ‘olfaction disorders’ or ‘taste disorders’, but we could not find any article. To the best of our knowledge, this is the first case report of duloxetine causing dysosmia and subsequent dysgeusia.

The specific mechanisms of drug-induced taste and smell disorders elicited by duloxetine and by the other drugs remain unknown. Moreover, the possible drug interactions with duloxetine related to concomitant taste and/or smell dysfunction, particularly ongoing amlodipine^6^ and lansoprazole^5^ which were prescribed before and after admission for this patient and, which have been associated with the above side effects, could not be excluded. Two possible hypotheses were proposed by Tuccori *et al*^5^ as a mechanism of drug-induced taste and/or smell alternations. The primary mechanisms resulted from a direct action of the drug; drug–receptor interaction, disturbance of action potential propagation in cell membranes, alteration of the neurotransmitter function and changes in connections between neural networks in brain regions associated with sensory coding and modulation. The secondary mechanisms include limiting the access of chemicals to sensing receptors, and changing the chemical or ionic milieu in the environment of sensing receptors.

By comparing olfactory acuity tests before and after discontinuing duloxetine (figure 1), it was observed that duloxetine impaired all olfactory detection thresholds to <2 levels, except for those of the rotten egg smell. This explains why the patient could smell rotten eggs at the coffee shop. In this patient, duloxetine decreased olfactory recognition thresholds, particularly of rose and rotten egg smells.

## Conclusions

This is the first report of duloxetine causing dysosmia and subsequent dysgeusia. Because duloxetine may impair olfactory cognitive function rather than olfactory detection threshold, close attention must be paid to the decreased sense of smell when duloxetine is prescribed.

**Learning points**

- This is the first report of duloxetine causing dysosmia and subsequent dysgeusia.
- This adverse effect was reversible because both dysosmia and dysgeusia were improved when duloxetine was discontinued.
- Close attention must be paid to the decreased sense of smell and/or taste when duloxetine is prescribed.
